# Formulation of Budesonide-Loaded Polymeric Nanoparticles into Hydrogels for Local Therapy of Atopic Dermatitis

**DOI:** 10.3390/gels10010079

**Published:** 2024-01-20

**Authors:** Marta Slavkova, Christophor Lazov, Ivanka Spassova, Daniela Kovacheva, Ivanka Pencheva-El Tibi, Denitsa Stefanova, Virginia Tzankova, Petar D. Petrov, Krassimira Yoncheva

**Affiliations:** 1 Faculty of Pharmacy, Medical University of Sofia, 1000 Sofia, Bulgariaitibi@pharmfac.mu-sofia.bg (I.P.-E.T.); denitsa.stefanova@pharmfac.mu-sofia.bg (D.S.); vtzankova@pharmfac.mu-sofia.bg (V.T.); 2 Institute of General and Inorganic Chemistry, Bulgarian Academy of Sciences, 1113 Sofia, Bulgaria; ispasova@svr.igic.bas.bg (I.S.); didka@svr.igic.bas.bg (D.K.); 3 Institute of Polymers, Bulgarian Academy of Sciences, Akad. G. Bonchev Str. 103A, 1113 Sofia, Bulgaria; ppetrov@polymer.bas.bg

**Keywords:** budesonide, nanoparticles, Eudragit L100, hydrogels, atopic dermatitis

## Abstract

Budesonide is a mineral corticoid applied in the local therapy of pediatric atopic dermatitis. Unfortunately, its dermal administration is hindered by the concomitant adverse effects and its physicochemical properties. The characteristic pH change in the atopic lesions can be utilized for the preparation of a pH-sensitive nanocarrier. In this view, the formulation of Eudragit L 100 nanoparticles as a budesonide delivery platform could provide more efficient release to the desired site, improve its penetration, and subsequently lower the undesired effects. In this study, budesonide-loaded Eudragit L100 nanoparticles were prepared via the nanoprecipitation method (mean diameter 57 nm, −31.2 mV, and approx. 90% encapsulation efficiency). Their safety was proven by cytotoxicity assays on the HaCaT keratinocyte cell line. Further, the drug-loaded nanoparticles were incorporated into two types of hydrogels based on methylcellulose or Pluronic F127. The formulated hydrogels were characterized with respect to their pH, occlusion, rheology, penetration, spreadability, and drug release. In conclusion, the developed hydrogels containing budesonide-loaded nanoparticles showed promising potential for the pediatric treatment of atopic dermatitis.

## 1. Introduction

Atopic dermatitis is a chronic, relapsing inflammatory skin disease. It has three stages: infantile, childhood, and adult. The onset could be as early as birth and can manifest with erythematous papules and vesicles on the cheeks, forehead, and/or scalp. It has a high prevalence and affects 15% to 30% of children [1]. The disease’s pathogenesis is related to genetic predisposition, environmental factors, and immune dysregulation [2]. It is characterized by increased transepidermal water loss due to barrier dysfunction and a pH increase of up to 6 or even higher [3]. Therapy for atopic dermatitis depends on the manifestation of the disease, and in severe cases, it may require a systemic remedy. In mild to moderate conditions, usually topical therapies are sufficient for disease management [4]. Furthermore, topical application is a convenient and affordable method of administration with minimal systemic toxicity [5]. The pharmacological topical treatment consists mainly of glucocorticoids, calcineurin inhibitors, or topical crisaborole [6]. Current therapeutic strategies are focused on reducing inflammation, restoring the skin barrier, and antibacterial therapy [7].

Corticosteroids are used as a first-line treatment for many dermal conditions, from pruritic lesions to atopic dermatitis or psoriasis. They have anti-inflammatory, immunosuppressive, anti-proliferative, and vasoconstrictor effects [8]. Even though they have been intensively applied topically, they have been associated with local or systemic adverse effects such as cutaneous atrophy, telangiectasia, striae, skin infections, and hypothalamic-pituitary-adrenal axis suppression [9]. This is a drawback in their application, especially for long-term therapies for chronic conditions such as atopic dermatitis.

Budesonide is a potent synthetic nonhalogenated representative of the corticosteroid family with predominantly anti-inflammatory activity and a weak mineralocorticoid [10]. It is applicable in the inhaled therapy of asthma [11] and the targeted oral treatment of ulcerative colitis [12]. There are recent reports about the use of budesonide in the management of pediatric atopic dermatitis [4]. However, budesonide has poor aqueous solubility [13] and a partition coefficient (log P) of 2.32 [14]. These characteristics make it an unattractive drug for the dermal route of application, and different strategies have been proposed to improve its skin delivery, e.g., cyclodextrin inclusion complex-based hydrogels [4], PLGA-PVA nanoparticles [10], and PEO-PCL-PEO triblock nanoparticles [15].

Nowadays, scientific efforts are directed toward the development of innovative formulations like nanoparticles, liposomes, microemulsions, etc. for enhanced delivery of drug molecules into the skin [7]. The nanotechnological approach can boost the therapy of skin disorders. The penetration and transport of drugs from nanoparticles can be modified by the different chemical properties of the polymer used, the encapsulation mechanism, the size of the nanoparticles, and the viscosity of the formulations. The loading of the drug in nanocarriers improves the solubility of highly hydrophobic drugs, provides sustained and controlled release, increases drug stability, and provides site-specific delivery. Therefore, the adverse effects can be diminished [10]. Polymeric nanoformulations are sub-micrometric colloidal drug carriers prepared by biocompatible and biodegradable polymers. They vary in composition and structure and include such nanosystems as nanocapsules, nanospheres, nanofibers, etc. [7]. Natural and synthetic polymers can be used in their preparation. The second group is characterized by high purity and batch-to-batch reproducibility and is therefore suitable for more consistent drug release profiles. Some typical examples of synthetic polymers used for nanoparticle preparation are poly(lactic-co-glycolic acid), tyrosine-derived triblock polymer, poly(ε-caprolactone), and others [16].

Polymeric nanoparticles can be prepared by various methods, which can be generally classified as the application of preformed polymers or the direct polymerization of monomers. There are various techniques applied for the formation of nanoparticles with preformed polymers, such as solvent evaporation, salting-out, dialysis, supercritical fluid technology, and others [17]. Eudragit^®^ is a manufactural name for a diverse range of synthetic polymethacrylate-based copolymers. They can be commercialized with different acidic or alkaline end groups, allowing pH-dependent drug release. Eudragits are functional polymers widely used in the development of polymeric nanoparticles. They have the potential to encapsulate and increase the solubility and bioavailability of poorly soluble drugs [18], as well as control drug delivery [3]. Eudragit^®^ L100 is an anionic representative with a mean molecular mass of approximately 135,000 Da and an apparent viscosity of 50–200 mPas [19]. It is soluble at pH ≥ 6 and is generally used in the preparation of enteric coatings. A current review of its applications shows that Eudragit^®^ L100 can be utilized in the preparation of microspheres, microsponges, nanoparticles, liposomes, tablets, etc. in order to achieve sustained release or bioavailability improvement [20]. Nanoparaticles based on Eudragit L100 were also proposed for dermal drug delivery [21]. It has also been suggested that those nanoparticles possess negative zeta potential and remain on the epidermis surface, limiting systemic absorption as well as side effects, which could be especially useful in the pediatric population [22].

Topical corticosteroids are available in different conventional dosage forms, including creams, ointments, gels, sprays, foams, and others [8]. The main barrier for the topical and transdermal routes of administration happens to be the stratum corneum (SC) layer of the skin. It has a two-compartment structure, often referred to as a “brick and mortar system.0”. The corneocytes are stacked in up to 20 layers and play the physical barrier role of SC. The spaces between them are occupied by mortar lipids. This is a complex mixture of around 13 lipid types, including ceramides, cholesterol, and free fatty acids, which play the permeability barrier role of SC [23]. The substances that are capable of diffusion through the intracellular route of the SC are small (molecular weight ≤ 400 Da) and lipophilic in nature (log P > 3) [24]. The main issue regarding the application of the classic semi-solid formulations is the enhancement of drug penetration and the simultaneous minimization of the risks of percutaneous absorption. The choice of vehicle can significantly affect the potency of the corticosteroid applied. Ointments, for example, exert more pronounced occlusion, which promotes their absorption and reach into the bloodstream [25]. In addition, the pediatric population, which is the most common end user of topical steroid therapy, is characterized by considerable differences in skin structure and thickness. The drug permeability is significant in children due to the thinner skin and the high skin-to-body-weight ratio. Thus, topical steroid application in children is prone to more systemic side effects such as growth retardation, Cushing disease, hyperglycemia, Addisonian crises upon cessation, and others [26]. Therefore, significant attention should be paid to the choice of vehicle for corticosteroid topical delivery. Simultaneously, more effective therapy regarding the dose is needed, with limited effect on the depth of penetration. 

Hydrogels present one of the most intensively used semisolid forms due to their excellent biocompatibility, solubility in water, and structural and viscoelastic resemblance to the cell membrane [27,28]. In addition, they are more cosmetically appealing, they do not cause skin maceration or folliculitis, and they can be applied to the scalp [25]. In the light of atopic dermatitis treatment, there is data suggesting that hydrogels are the most preferable dosage form [29]. The most recent studies have pointed towards the preparation of so-called novel hydrogels, which consist of novel formulations such as nanoparticles, nanoemulsions, microemulsions, liposomes, etc. [5]. These dosage forms provide the opportunity to resolve some of the issues of drug delivery to the skin as well as being capable of controlling the drug release. Various polymers could be used for hydrogel preparation, including natural ones (such as chitosan [30,31], hydroxyethylcellulose [32], and hyaluronic acid [33]) or synthetic ones (such as carbomer [34], pluronic [27], and polyvinyl alcohol [35]). The choice of gelling agent can affect the properties of the prepared hydrogel and the expected drug behavior. Even though carbomer is one of the most widely used gelling agents for semisolid formulations, its gelation is pH-dependent and occurs in a neutral medium [36]. This could be inappropriate for pH-dependent Eudragit^®^ L100 nanoparticles. Another considerably universal gelling agent for various routes of application are Pluronic derivatives [37]. Pluronics are water-soluble non-ionic triblock copolymers (PEO-PPO-PEO) of varying numbers of polyethylene oxide (PEO) and polypropylene oxide (PPO) units. Depending on the size of the blocks and molecular weight, different grades of Pluronic copolymers exist. The PEO and PPO blocks determine their amphiphilic structure, which allows micelle formation for the solubilization of lipophilic drugs. Depending on the type and concentration, a thermo-reversible gelation can be observed [37]. The most common representative applied as gelation aid is the hydrophilic Pluronic^®^ F127. Its ease of gelation and biocompatibility make it very suitable for semisolid topical formulations. A disadvantage is its relatively low mechanical strength. On the other hand, methylcellulose is a cellulose derivative with excellent biocompatibility properties. It is the simplest ether derivative, with methyl groups substituting the hydroxyl ones at the C-2, C-3, and/or C-6 positions. There are a lot of commercial grades of methylcellulose, varying the degree and localization of substitution. Methylcellulose hydrogels have been exploited for dermal, ocular, vaginal, rectal, and oral drug delivery [38]. Therefore, gelling agents from two different groups were chosen in the current study for comparison purposes. The consistency of the hydrogels and their spreadability are important characteristics that provide information regarding the application or delivery of a desired drug dose to the skin and the ease of gel application. These properties significantly influence the patient’s preference for the respective semisolid formulation [39].

The use of corticosteroids in the pediatric population should be considered with care, as these patients possess a higher propensity to develop adverse actions due to a higher surface area-to-body weight ratio and fragile skin [40]. A formulation of budesonide in an appropriate delivery system capable of providing efficient treatment of early forms of atopic dermatitis with limited side effects is a very attractive approach. The prolonged release achieved with the help of nanocomposites can overcome issues regarding systemic absorption through topical administration. Furthermore, a semisolid formulation could be suitable for easy application, a longer stay on the affected area, and a possible hydration effect. Thus, the aim of the current study is to develop and characterize Eudragit^®^ L100-based nanoparticles loaded with budesonide for pH-sensitive delivery of the drug. Furthermore, the drug-loaded nanoparticles were formulated into two types of semisolid hydrogels as a final dosage form for the therapy of atopic dermatitis.

## 2. Results and Discussion

In the present study, budesonide is encapsulated into Eudragit L100 nanoparticles that are further formulated in hydrogel dosage form. The scientific rationale is to combine the pH-dependent budesonide delivery via Eudragit L100 nanoparticles with the hydration ability of methylcellulose or F127 hydrogels as a final dosage form.

### 2.1. Preparation and Characterization of the Nanoparticles

**Figure 1:**
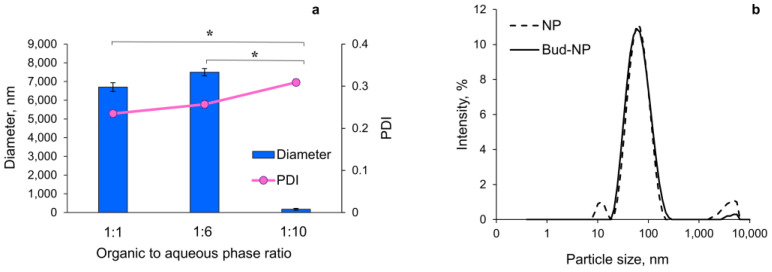
Mean diameter and polydispersity index with standard deviation (*n* = 3) of the particles prepared at different ratios between the ethanol and the aqueous phase (**a**) and histogram of particle size distribution by intensity of the optimized empty (NP) and budesonide-loaded nanoparticles (Bud-NP) (**b**) (significance level * *p* < 0.05).

Eudragit L100 nanoparticles were successfully prepared by the nanoprecipitation technique. Eudrgait L 100 and budesonide were dissolved in ethanol, and their solution was slowly precipitated via mixing with a 0.25% aqueous solution of PVA as a non-solvent. During this mixing, rapid diffusion of the ethanol occurs in the water, which is accompanied by reduced interfacial tension and the formation of small droplets of the polymer and drug. Upon ethanol evaporation, nanoprecipitation occurs [17]. It appeared that the pH of the aqueous PVA-phase was a crucial factor in the preparation of particles with a size on the nanoscale. The medium diameter of the particles obtained with PVA-phase at pH 4.0 was approximately 6268 nm, whereas those prepared at pH 5.0 had an average diameter less than 60 nm. Furthermore, the ratio between both the organic and aqueous PVA-phases also influenced the particle size. Figure 1a shows the three ratios between both phases that were evaluated (1:1, 1:6, and 1:10, *v*/*v*). The optimal ratio between the ethanol and the aqueous phase was determined to be 1:10, since only at this ratio was the size of the particles at the nanoscale. As shown, the polydispersity slightly increased at this ratio but still indicated a narrow size distribution. The results from the dynamic light scattering analysis (DLS) for the optimized batch are presented in Figure 1b. It can be seen that there is no significant difference between the size of the empty (57.2 nm) and the drug-loaded nanoparticles (55.8 nm). Both particle samples showed a narrow particle size distribution, with PDI equal to 0.309 and 0.219 for the NP and Bud-NP, respectively. Similar results were observed by other studies [41].

**Figure 2:**
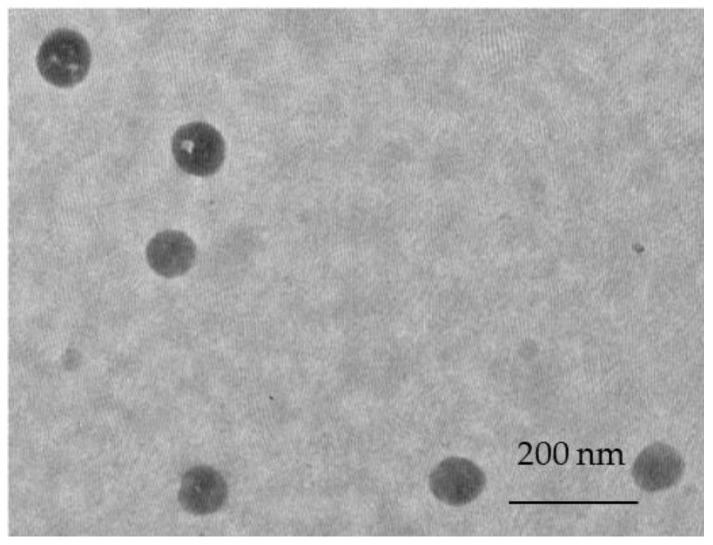
Transmission electron microscope image of the optimized nanoparticle batch.

Important information regarding nanoparticle colloidal stability can be provided by investigating their zeta potential. According to literature data, polymeric nanoparticles are considered stable if their absolute value of zeta potential is equal to or greater than 30 mV [42]. All batches of the prepared nanoparticles were characterized with similar values ranging from −30 mV to −32.7 mV. The negative zeta potential could be explained by the presence of carboxylic groups in the polymer carrier on the nanoparticle surface. Similar results regarding Eudragit L100 nanoparticles can be found in the literature [3,43,44]. The morphology of the prepared nanoparticles was characterized by TEM. The micrographs are presented in Figure 2. It can be seen that the nanoparticles were spherical in shape, and the observed diameter was correlated with that found by DLS.

**Figure 3:**
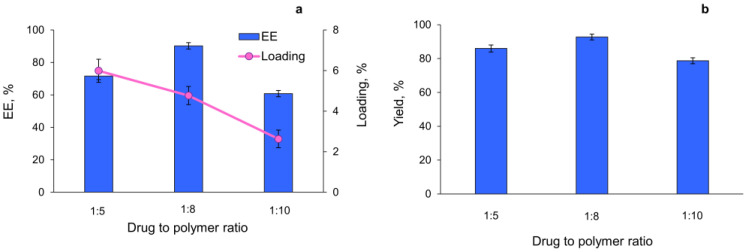
Influence of the ratio between the drug and the polymer on encapsulation efficiency, drug loading (**a**), and nanoparticle yield (**b**).

The encapsulation efficiency was investigated in the case of different ratios between the drug and the polymer (correspondingly 1:5, 1:8, and 1:10, wt/wt). The results from the different batches are statistically different (*p* < 0.005). The results showed that encapsulation efficiency was paramount at a ratio of 1:8, achieving approximately 90% (Figure 3a). Similarly, the yield of the obtained lyophilized nanoparticles was highest at the same ratio (Figure 3b). The loading degree at a ratio of 1:8 was slightly lower than that obtained at a ratio of 1:5 (Figure 3a). Thus, taking into consideration the higher encapsulation efficiency and higher yield, the ratio 1:8 was selected as optimal, and all further tests were performed with these nanoparticles.

**Figure 4:**
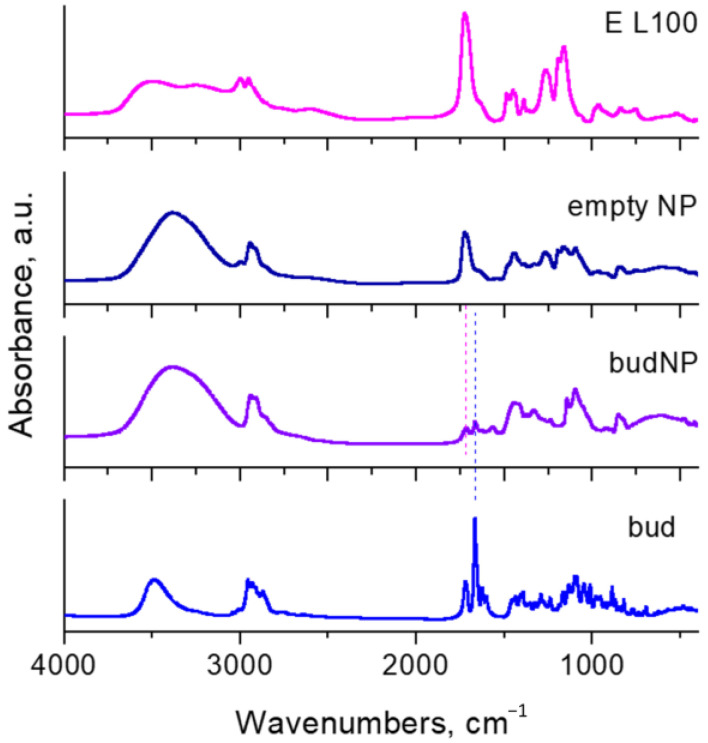
FTIR spectra of budesonide (Bud), budesonide-loaded nanoparticles (Bud-NP), empty nanoparticles (NP), and Eudragit L100 (E L100).

Figure 4 presents the FTIR spectra of budesonide, Eudragit L100, empty nanoparticles, and budesonide-loaded nanoparticles. The spectrum of budesonide consists of lots of well-resolved absorption peaks, which could be assigned as follows: a peak in the region 3600–3350 cm^−1^ due to OH-group stretching; peaks in the region 3000–2850 cm^−1^ due to the stretching of C−H bonds; peaks at 1721 cm^−1^, 1666 cm^−1^ and 1622 cm^−1^, attributed to stretching vibrations of C=O (carboxylic), conjugated C=O stretching, and C=C bonds, respectively. The Eudragit L100 spectrum represents a broad band in 3700–3080 cm^−1^ of stretching of the OH-group, which overlaps partially with the peaks of the C−H stretch in the 3060–2870 cm^−1^ range. A peak with high intensity at 1722 cm^−1^ is assigned to C=O ester stretching with a shoulder at 1620 cm^−1^. The peaks of C−H bending vibrations are found in the region 1380–1470 cm^−1^. The spectrum of the empty nanoparticles consists of the same peaks as this of Eudragit L100, with a slight difference in their intensities. The spectrum of the drug-loaded sample (Bud-NP) is characterized by the same peaks observed in the spectrum of the empty nanoparticles, accompanied by a noticeable additional peak at 1664 cm^−1^ characteristic for the budesonide, which confirms the loading of budesonide into the nanoparticles formed. 

**Figure 5:**
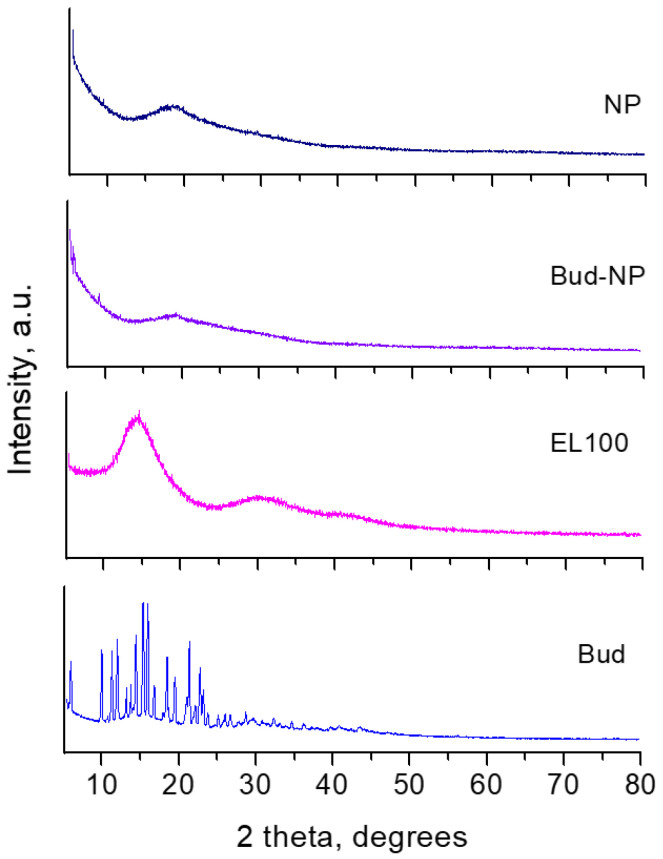
Powder XRD patterns of budesonide (Bud), budesonide-loaded nanoparticles (Bud-NP), empty nanoparticles (NP), and Eudragit L100 (E L100).

Part of the XRD patterns of budesonide, Eudragit L100, empty nanoparticles, and budesonide-loaded nanoparticles are presented in Figure 5. The pattern of budesonide represents a well-crystalline compound with two epimers (22R and 22S) [45]. Our detailed examination also revealed that it consists of patterns of the 22R and 22S epimers. The mass ratio of 3:1 (75% and 25% for 22R and 22S epimers, respectively) was calculated by comparing the total intensity of the peaks connected to both phases with the assumption of their equal density. Both epimers were found to crystallize in orthorhombic Space Group P2_1_2_1_2_1_, and their unit cell parameters are calculated by our experimental data as follows: for 22R a = 8.516(4) Å, b = 9.185(3) Å, c = 28.87(1) Å; for 22S a = 8.449(2) Å, b = 9.127(2) Å, c = 20.099(9) Å. It is worth mentioning that they are very close to those reported in the study of Albertsson et al. [45]. The XRD pattern of Eudragit L100 shows typical amorphous humps at around 2θ = 15° and 30°, as it was observed previously [46]. The well-visible shift of the maximum of the first amorphous peak from 2θ = 15° to 18.5° upon the formation of empty nanoparticles can be seen. The shift indicates that, in the presence of PVA as a stabilizer, the characteristics of the encapsulated Eudragit L100 differ from those of the bulk Eudragit L100. This is a common feature of nanoparticles. In particular, the surface layer tends to have many structural defects, resulting in different types of bonding and coordination of the atoms compared to the bulk carrier [47]. The loaded sample shows the same amorphous peak as in the empty nanoparticles and some small crystalline peaks at around 2θ = 6° and 10° indexed as (002) and (011) peaks of budesonide. The results confirm the successful loading of budesonide into Eudragit L100 nanoparticles. 

### 2.2. Cytotoxicity Evaluation of HaCaT Cells

Cell cytotoxicity assays represent one of the most frequently used in vitro bioassay methods for predicting the toxicity and irritating side effects of drugs and medical devices; thus, it is important to study thoroughly the response of cell mechanisms upon exposure to different compounds [48]. Cultured human keratinocytes offer a means to predict dermal irritancy resulting from exposure to various substances in humans [49,50]. Keratinocytes, being the first living cells that come into contact with externally applied compounds, represent a biologically relevant target for assessing skin irritants. However, primary keratinocyte cultures have inherent limitations, including limited and variable availability of source material and varying susceptibility to irritants with the number of passages. To overcome these challenges, HaCaT cells were employed as a model. These non-tumorigenic, spontaneously immortalized keratinocyte cells offer a nearly limitless supply of identical cells, thereby ensuring high levels of reproducibility within and between laboratories [50]. Furthermore, it is worth noting that in vitro cytotoxicity data obtained from the human keratinocyte line (HaCaT) closely correlates with in vivo data [51].

**Figure 6:**
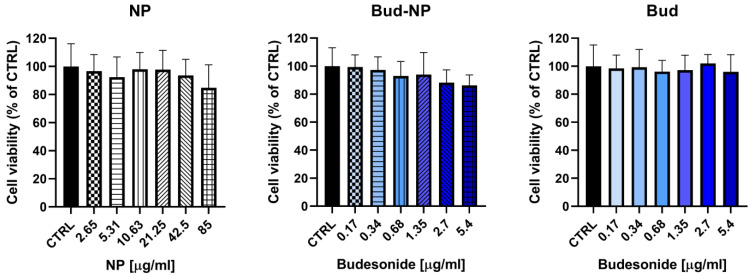
Cytotoxicity on HaCaT cells measured by MTT assay of pure budesonide (Bud), budesonide loaded in the nanoparticles (Bud-NP), and empty nanoparticles (NP). The results are expressed as means ± SD of triplicate assays (*n* = 3). All groups were compared statistically vs. untreated controls by one-way ANOVA with Dunnet’s post hoc test.

Therefore, we evaluated the potential cytotoxic effects of pure budesonide, budesonide loaded into the nanoparticles, and empty nanoparticles on the viability of the human keratinocyte line HaCaT. A colorimetric assay measuring the capacity for viable cells to metabolize a tetrazolium colorless salt to a blue formazan (MTT assay) was used as an indirect measurement of cell viability to predict skin irritancy. After 24 h of treatment, the empty nanoparticles (NP) at concentrations ranging from 2.65 to 85 μg/mL did not decrease cell viability and showed no toxic effects. Furthermore, both pure budesonide (ranging from 0.17 to 5.4 μg/mL) and budesonide loaded into the nanoparticles (corresponding concentrations) did not exhibit a statistically significant decrease in keratinocyte viability, as shown in Figure 6. In the tested concentrations, both samples demonstrated no in vitro toxicity and a favorable safety profile in the human keratinocyte HaCaT cell line.

### 2.3. Preparation and Characterization of the Hydrogels

The nanoparticle dispersions can be easily removed from the skin and exert limited contact with the affected area. Thus, in order to improve the retention of the drug at the site of application, the nanoparticles were incorporated into two types of hydrogels. Furthermore, the high water content of hydrogels would provide hydration for the atopic skin. The latter makes hydrogels very appropriate topical dosage forms since the disrupted barrier function of atopic skin allows transepidermal water loss. The hydrogels are prepared by simple gelling of nanoparticle dispersions with methylcellulose or Pluronic F127 (further referred to as F127). The gelling agent selection was based on their frequent use, biocompatibility, and generally regarded as safe (GRAS) status [52,53]. Light microscopic observations showed that the incorporation of the nanoparticles within both hydrogels did not lead to any changes in their appearance or stability. All gels maintained a homogeneous, transparent appearance with no visible aggregates upon nanoparticle incorporation. In addition, the pH of the prepared hydrogels was determined with or without the presence of nanoparticles. The F127 and methylcellulose plain gels had pH equal to 5.19 and 5.26, respectively. The results suggested that the semisolid vehicles are appropriate for the incorporation of the Eudragit L100 nanoparticles. The incorporation of the nanoparticles leads to a slight but insignificant decrease in the pH values (5.11 and 5.18, respectively). This can be attributed to the presence of PVA as a stabilizer for the nanoparticles. The data suggests the suitability of the proposed formulations for dermal application [54].

Preliminary dynamic rheological tests of methylcellulose- and Pluronic- based samples revealed a significant difference in the elastic properties of materials (Table 1). The hydrogel formed by F127 was much more elastic than the methylcellulose hydrogel (MC). At first glance, one of the reasons for the huge difference in the elastic modulus (G′) of F127 and MC hydrogel carriers might be their different concentrations. More precisely, at the given concentration, the plain MC sample was in the form of a highly viscous solution (G″ > G′), which formed a soft gel upon adding the nanoparticles. On the other hand, the F127 system exhibited the typical behavior for hard gels (G″ >> G′) with and without NPs. It should also be noted that the gelation of the two polymers in aqueous media occurs by different mechanisms. Above certain critical concentrations and temperatures, the macromolecules of MC tend to intertwine, and some junction pints are formed to produce a weak physical hydrogel [55]. In contrast, under the reported experimental conditions, F127 macromolecules are self-assembled into nanosized micelles, which are closely packed into a three-dimensional network structure. Such material behaves as a hard gel [56].

Embedding the empty and budesonide-loaded nanoparticles into the hydrogel matrix resulted in increased elastic modulus and complex dynamic viscosity (η*) (see Table 1). The reinforcing effect of the nanoparticles can be explained by the fact that the NPs comprise a polymethacrylate derivative, which makes them more rigid than the hydrogel matrix.

**Figure 7:**
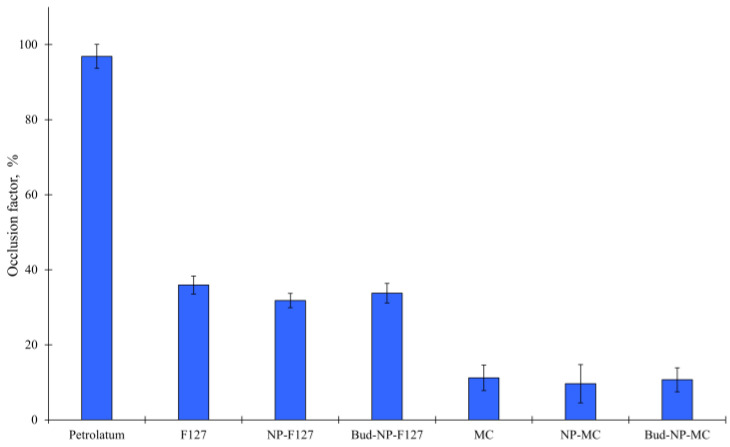
Occlusion factor for the plain methylcellulose (MC) and Pluronic F127 (F127) hydrogels and the same hydrogels containing the empty (NP-F127 and NP-MC) or drug-loaded nanoparticles (Bud-NP-F127 and Bud-NP-MC). Petrolatum was used as a positive control.

The investigation of the occlusive properties of the selected gel bases was evaluated, taking into consideration their administration to atopic lesions. The occlusive properties of both hydrogels are compared to those of petrolatum, which is well known for its high occlusion [57]. The hydrogels are preferable semisolid vehicles for dermal delivery due to their more appealing properties and the reduced occlusive effect they possess [28]. Indeed, our study reveals that both hydrogels have a lower occlusion factor than petrolatum (Figure 7). The observed occlusive effect is due to the gelling agent present in the formulation, which tends to form a thin film on the surface, thus preventing water evaporation [58]. The lower occlusive effect compared to petrolatum is attributed to the hydrophilic properties of the gels. This result indicates that the hydrogels will ensure breathability during skin treatment. Thus, the combination of breathability and hydration ability of the developed hydrogels could be considered important parameters for effective healing of atopic lesions [59]. Further, the F127 gel showed more pronounced occlusion compared to the methylcellulose gel. This may be attributed to the lower concentration at which methylcellulose is used for the gelation. This is probably not the only reason since there is data in the literature that 0.5%–0.8% Carbopol-based gels showed an occlusive factor similar [60] or even higher [58] to the one of plain methylcellulose hydrogel in our study. Probably, the difference in the chemical structure of the gelling agents affects film formation and the prevention of water evaporation.

Further, the presence of nanoparticles was evaluated in terms of their effect on occlusive properties. It is well known that lipid nanoparticles exhibit skin occlusive effects [58,60]. There are limited data characterizing the occlusion of polymeric nanoparticles. Thus, in the present study, we investigated whether the embedment of Eudragit L100 nanoparticles within two different hydrogels affects their prevention of water evaporation. The results showed that the incorporation of empty or drug loaded nanoparticles did not significantly alter the occlusion factor of the parent hydrogels. The retention of water could be useful in terms of the effectiveness of drug delivery as it could hydrate the stratum corneum [61] and also ameliorate the atopic skin condition [62]. 

**Figure 8:**
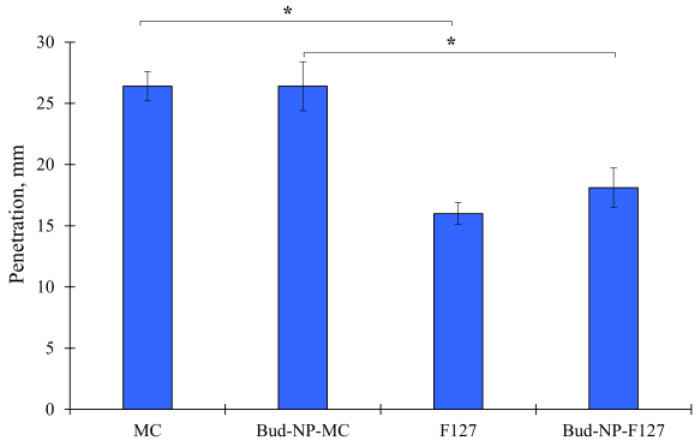
Penetration of plain hydrogels (MC and F127) and hydrogels containing budesonide-loaded nanoparticles (Bud-NP-MC and Bud-NP-F127); mean ± SD, *n* = 3; (* significant difference at level *p* < 0.05).

The penetration and spreadability of the prepared hydrogels were also evaluated in order to provide some information regarding their ease of application. As can be seen in Figure 8, the addition of nanoparticles within the hydrogels is not related to a significant alteration of the depth of penetration. It can be seen that the F127-based gel shows a statistically lower depth of penetration compared to the methylcellulose gel (*p* = 0.012, one-way ANOVA). According to the rheology, it could be due to the closely packed three-dimensional network structure of this hydrogel compared to the weak physical hydrogel of methylcellulose. Similar values for the depth of penetration in F127 gels have been reported by other working groups [63].

**Figure 9:**
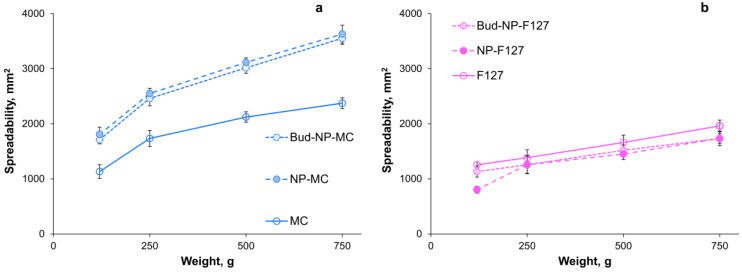
Extensiometric profiles of plain methylcellulose (MC) (**a**) and F127-based gels (**b**) and the same hydrogels containing empty (NP-MC and NP-F127) or drug-loaded nanoparticles (Bud-NP-MC and Bud-NP-F127). Mean ± SD, *n* = 3.

The results from the spreadability test for the plain and nanoparticles containing hydrogels are presented in Figure 9. It can be seen that the F127 gels are less spreadable than the ones with methylcellulose as a gelling agent. This can be attributed to the higher concentration used for gelation. Such results can be found in the literature [64]. According to the literature, methylcellulose solutions with a concentration of about 1% or less show thermogellation above 30 °C, depending on the molecular weight of the used methylcellulose [38]. Furthermore, the increase in molecular weight and concentration leads to gelation at a lower temperature [65], and typically hydrogels are formed at room temperature at a concentration of 3–6% [66]. In our study, a high-molecular weight methylcellulose was used at a concentration of 4%, leading to the formation of gel at room temperature with a spreadability factor of 5.95 mm^2^/g. The incorporation of the Eudragit L100 nanoparticles in the methylcellulose hydrogels resulted in an increase in hydrogel spreadability, as shown in Figure 9a, and the corresponding spreadability factors are 9.09 mm^2^/g and 8.91 mm^2^/g for the NP-MC and Bud-NP-MC samples, respectively. Since Zilberman et al. reported a decrease in surface tension for the mixtures of cellulose derivatives and PVA [67], we suggest that the presence of PVA in nanoparticle dispersion may contribute to the larger spreadability of the hydrogels containing the nanoparticles.

In the case of F127-based gels, no significant difference is observed between the plain and composite gels, as shown in Figure 9b. Only a slight decrease in the spreadability factor is evidenced for the hydrogel loaded with the empty nanoparticles (4.23 ± 2.02 mm^2^/g) compared to the empty hydrogel (5.49 ± 3.55 mm^2^/g) and the hydrogel formulated with budesonide-loaded nanoparticles (4.96 ± 3.21 mm^2^/g). Similar decrease in spreadability was observed for F127-based gels loaded with different types of nanoparticles [68].

Thus, the different effects of nanoparticle incorporation in the two types of hydrogels could be explained by the different mechanisms of gelation for F127 and methylcellulose. According to most recent literature studies, methylcellulose forms gel based on the fibril theory [53,69]. The coiling of the fibrils is more pronounced at lower pH values as opposed to higher pH values, and the viscosity is correspondingly lower as there is limited possibility for polymer-polymer interaction [70]. In the present study, the incorporation of the nanoparticles leads to a slight reduction of the pH. At the same time, F127 gelation is due to the very tight packing of the formed micelles and their overlaying [37,71]. The enthropy is determining the gelation process [37], and probably the nanoparticles do not affect it. 

**Figure 10:**
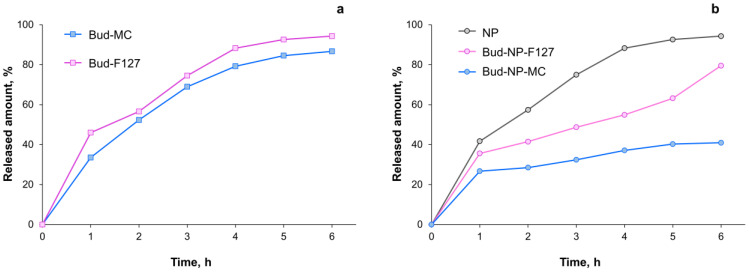
In vitro drug release from the hydrogels containing non-encapsulated budesonide (**a**), nanoparticles, and hydrogels with nanoparticles (**b**) in a buffer medium (pH 5.5).

The release profiles of the free drug from the two hydrogels as well as budesonide-loaded nanoparticles (Bud-NP) and their corresponding hydrogels are shown in Figure 10. The drug release from the nanoparticle dispersion fits the best Higuchi release kinetics (Table 2). It can therefore be expected that budesonide release is diffusion-driven through the undissolved Eudragit L100 matrix nanoparticles. Similar results for the release of Eudragit-based polymeric nanoparticles have been reported by other researchers [72,73]. Similarly, the Higuchi model best fits the release of non-encapsulated budesonide from the two hydrogels (Table 2). As shown, with values for the diffusional exponent *n* > 0.5 (Korsmeyer-Peppas model), a non-Fickian diffusion controlled the release from the hydrogels, whereas quasi-Fickian diffusion could be considered in the case of nanoparticles (*n* < 0.5).

Comparing the release from both hydrogels containing non-encapsulated (Figure 10a) or encapsulated drugs (Figure 10b), it can be concluded that the methylcellulose gel is characterized by a slower and incomplete release as opposed to the F127. The drug release from the NP-loaded F127 gel follows zero-order (Table 2). Such behavior is observed by other researchers as well [74]. It is due predominantly to the F127 dissolution in the medium [75,76]. In the case of methylcellulose gel, the swelling of the polymer retards the drug release. Furthermore, the investigation of the release kinetics suggests a first-order pattern. This is in accordance with previous data suggesting that the polymer itself may retain the drug [77]. Another study comparing the release of free drugs from methylcellulose and poloxamer gel showed the slowest release from the methylcellulose gel, even though the viscosity was lower than the Pluronic F127 one [78]. The authors explain these findings due to the interaction between the polymer and drug. Another study points towards the significance of drug-polymer interactions rather than the viscosity or concentration of the polymer [79]. In the current study, probably the Eudragit nanoparticles stabilized with PVA interact with methylcellulose but not with Pluronic F127. These assumptions are also supported by the susceptibility of the methylcellulose gel’s spreadability and viscosity to the incorporation of the proposed nanoparticles. The calculation of the similarity factor between the two release profiles shows that they are not similar (f_2_ = 33.1). Possible reasons for the different effect that the gelling agent exerts on the release pattern are the surface active properties of F127 and its tendency to form micelles at concentrations above 0.725 wt% at 25 °C [80]. Therefore, in the case of F127 hydrogel, budesonide is probably solubilized, which enables the release process. Furthermore, the drug release from Pluronic F127 gel is governed by gel erosion and is not affected to a significant extent by drug diffusion [71]. Such an assumption is supported by the release kinetics findings in the current study.

## 3. Conclusions

Budesonide was successfully encapsulated in Eudragit nanoparticles (approximately 90% encapsulation efficiency) intended to provide local drug delivery at pH 5.5 and above, which is desired for atopic skin treatment. The nanoparticles possess appropriate physicochemical properties, particularly their small size and highly negative surface charge, that are prerequisites for improved penetration and colloid stability, respectively. Prolonged release was achieved, which could reduce the applied dose. The lack of irritancy of the prepared nanocarriers was demonstrated in vitro in the human keratinocyte cell line, HaCaT. Further, the budesonide-loaded nanoparticles were homogeneously embedded in two types of hydrogels, based on methylcellulose or Pluronic F127, able to provide ease of application and hydration ability to the topical formulation. Both hydrogels showed suitability for dermal application in terms of spreadability, penetration, pH, and occlusion properties. At the same time, the budesonide release from the F127 gel was more complete in the tested time frame, making it more practically applicable.

## 4. Materials and Methods

### 4.1. Materials

Budesonide, methylcellulose (Methocel 90HG), and ethanol (96%) were purchased from Sigma Aldrich; poly(methacrylic acid-co-methyl methacrylate) 1:1 (Eudragit^®^ L100) from Evonik Röhm GmbH (Darmstadt, Germany); polyvinyl alcohol (PVA 22000) from Fluka Chemie AG (Germany); and Pluronic F127 from BASF (Ludwigshafen, Germany). Distilled water was prepared in the laboratory. For HPLC analysis, acetonitrile and methanol HPLC grades were used from Fisher Chemical (Thermo Fisher Scientific Inc., Waltham, MA, USA). Dulbecco’s Modified Eagle’s Medium, MTT (3-(4,5-dimethylthiazol-2-yl)-2,5-diphenyltetrazolium bromide), fetal bovine serum, and L-glutamine were purchased from Sigma-Aldrich (Merck KGaA, Darmstadt, Germany). The Human immortalized keratinocyte cell line HaCaT (300493) was acquired from the CLS Cell Lines Service GmbH (CLS, Eppelheim, Germany).

### 4.2. Preparation of the Nanoparticles

**Figure 11:**
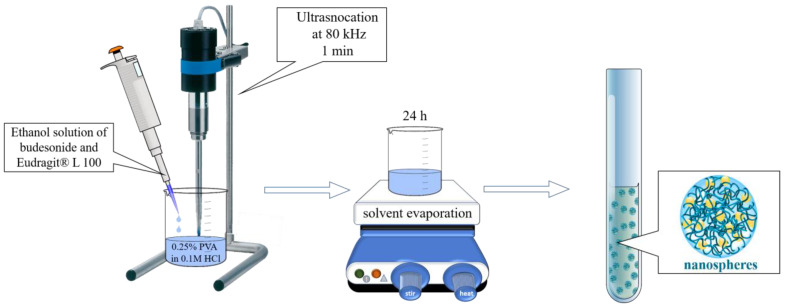
Schematic representation of the nanoprecipitation for nanoparticle preparation.

The nanoparticles were prepared by nanoprecipitation according to the procedure suggested by Sahle et al. [21], with some modifications as shown in Figure 11. First, PVA (0.25% wt/v) was dissolved in purified water, and the pH was adjusted to 5.0 by the addition of 0.1 N HCl. Eudragit^®^ L100 and budesonide were dissolved in 95% ethanol in different concentrations, giving the following ratios in regard to Eudragit^®^ L100: 1:5, 1:8, and 1:10 (wt/wt). Afterwards, the ethanol solution was added dropwise to the PVA while being sonicated at 80 kHz (Bandelin Sonoplus HD3100, Bandelin Electronics, Berlin, Germany) for 1 min. The sonication was continued for 1 more minute after the ethanol solution was added completely. Then, the resultant dispersion was left for 24 h under continuous stirring for ethanol evaporation. Upon the evaporation of the organic solvent, Eudragit^®^ L100 precipitates into nanoparticles stabilized by the non-ionic surfactant PVA. The prepared dispersion was filtered (0.45 µm), and the filters were rinsed with ethanol (50%). The encapsulation efficiency was determined based on the initial amount of budesonide (*Bud_total_*) and the amount found in the filter fractions (*Bud_filter_*). The following equation was used for the calculation:

(1) $$EE\%=\frac{Bud_{total}-Bud_{filter}}{Bud_{total}}.100$$

### 4.3. Determination of Nanoparticle Size, Polydispersity Index (PDI), and Zeta-Potential

Dynamic light scattering (DLS) was applied to investigate the particle size, polydispersity (PDI), and zeta-potential of the prepared empty and budesonide-loaded nanoparticles (Zeta-Master, Malvern Instruments, Worcestershire, UK). The measurements were performed in triplicate on the aqueous nanoparticle dispersions at 25 °C with a scattering angle of 90°. Transmission electron microscopy (TEM) was applied for the evaluation of nanoparticle shape and surface morphology (HR STEM JEOL JEM 2100, Tokyo, Japan).

### 4.4. X-ray Powder Diffraction Analysis (XRPD) and FTIR Spectrophotometry

The diffraction patterns of budesonide, budesonide-loaded nanoparticles, empty nanoparticles, and Eudragit L100 were collected from 5 to 80°2θ on a Bruker D8-Advance Diffractometer (Karlsruhe, Germany). CuKα radiation was used, and registration was performed by the LynxEye detector. The unit cell parameters were refined using the Topas 4.2 program, part of the Bruker software (Bruker AXS, Karsruhe, Germany).

The FTIR spectra of budesonide, budesonide-loaded nanoparticles, empty nanoparticles, and Eudragit L100 in KBr were recorded on a Thermo Nicolet Avatar 360 FTIR spectrometer (Thermo Fisher Scientific, Waltham, MA, USA), within the range 4000–400 cm^−1^ with a resolution of 2 cm^−1^.

### 4.5. Cytotoxicity Evaluation of HaCaT Cells

The cell line was cultured in 75 cm^2^ flasks in DMEM medium with glucose (4.5 g/L), to which 10% fetal bovine serum and 2 mM L-glutamine were added. The cells were maintained at a constant temperature of 37 °C within an environment comprising 5% CO_2_. When the cells reached approximately 80% confluence, a series of sequential steps were performed. The cells were first harvested using a trypsin/EDTA solution, after which they were precisely seeded into the central 60 wells of 96-well plates at a density of 5 × 10^4^ cells per milliliter. Subsequently, these plates were placed in an incubator and maintained at 37 °C with 5% CO_2_ for a period of 24 h. This process was meticulously repeated three times, utilizing cells from different passages, to ensure experimental consistency and reliability.

The MTT assay was employed to assess the cytotoxicity of the tested samples according to the previously described procedure [81]. The cells were treated with a reference solution of budesonide (0.17, 0.34, 0.68, 1.35, 2.7, and 5.4 μg/mL), dispersion of budesonide loaded nanoparticles (in the same concentrations), and dispersion of empty nanoparticles (from 2.65 μg/mL to 85 μg/mL). Each plate included control wells that contained only culture medium. After 24 h of treatment, the culture medium was aspirated and replaced with 100 μL of the MTT solution (5 mg/mL in phosphate-buffered saline) in each well. Subsequently, the plates were incubated for a period of 3 h, the cell culture medium was aspirated, and 100 μL of dimethylsulfoxide (DMSO) per well was added to dissolve the purple formazan product. This was achieved by gently shaking the plates for 10 min at room temperature. The absorbance of the resulting solutions was measured at 570 nm using a multiplate reader, Synergy 2 (BioTek Instruments, Inc., Highland Park, Winooski, VT, USA). 

For statistical analysis, GraphPad Prism 8 Software was utilized. The data underwent a one-way analysis of variance (ANOVA), which was followed by Dunnett’s multiple comparisons post-test. This post-test was employed to assess and compare differences between the control and treatment groups. A significance level of 0.05 was selected as the threshold for determining statistical significance in all the comparisons conducted.

### 4.6. Hydrogel Preparation

A hydrogel was proposed as a semisolid dosage form containing budesonide loaded nanoparticles. Two types of hydrogels were formulated using methylcellulose (MC) (4 wt %) and Pluronic F127 (F127) (25 wt %) as gelling agents. Pluronic F127 was dispersed in the medium, while methylcellulose was dispersed in the hot medium, and then they were both left to dissolve completely at 4 °C overnight. The gels containing budesonide-loaded nanoparticles were prepared in a similar manner, with the liquid medium being the nanoparticle-containing dispersion. In the case of methylcellulose, the different gelling agent concentrations were compensated by the addition of distilled water with a pH of 5.0. In this way, the budesonide concentration in both hydrogels was adjusted to 0.1 mg/g gel.

### 4.7. Appearance and pH of Hydrogels

All formulations were visually and microscopically investigated using a Leica DM750 light microscope equipped with Air Teach software (v.1.0.9874) (Heerbrugg, Switzerland). The pH of the formulations was determined by the potentiometric method with a pH meter (Hanna HI98100, Hanna Instruments Inc., Woonsocket, RI, USA). The investigated gel samples were diluted (1:4) with distilled water, mixed vigorously for 1 min, and the pH was recorded [82,83].

### 4.8. In Vitro Occlusion Test

The occlusive properties of the gels were tested in vitro based on the measurement of water evaporation in controlled environmental conditions, as proposed by Caldas et al. [84]. In brief, a beaker was filled with 25 mL of distilled water, covered with a Whatman cellulose filter (0.45 µm), and tightly sealed with Teflon tape. An equal amount of the tested gels was evenly spread on the surface of the filter paper (surface area: 13.84 cm^2^). The samples were accurately weighed and stored in a climate chamber (T = 32 ± 0.5 °C; RH = 50% ± 1%) in the dark. After 48 h, the samples were weighed again. The water loss of the sample (*L_S_*) was calculated based on the change in weight. The difference with the reference sample’s loss (*L_R_*) was used for the calculation of the occlusive factor (*F*).

(2) $$F\%=\frac{L_{R}-L_{s}}{L_{R}}.100$$

The reference sample was a beaker with plain filter paper on top. An occlusive factor of 100 means a maximal occlusive effect, while an occlusive factor of 0 means no occlusion [85].

### 4.9. Rheology, Spreadability, and Penetrometry of Hydrogels

Dynamic rheological measurements of hydrogels were carried out with a HAAKE MARS 60 rheometer in controlled deformation mode using a parallel plate sensor system (top plate diameter = 20 mm; gap = 1 mm). The elastic (G’) modulus was determined at 32 °C and constant deformation (γ = 0.01) in the 0.1–10 Hz frequency range. 

The spreadability test characterizing the rheological properties of the hydrogels was performed with the parallel plate method [86,87]. A circle with a diameter of 1 cm was marked on a glass plate, and a sample of 1 g of the tested gel was placed inside. A second glass plate with a known weight was set on top. Subsequent weights are placed on top every 5 min. The diameter (*d*) of the spread gel was measured and recorded after each weight (*W*). The results were plotted to obtain the extensiometric profiles of the samples. All measurements were performed in triplicate. The spreadability (*S*) and spreadability factor (*S_F_*) were calculated based on the following equations:

(3) $$S=\frac{d^{2}.\pi }{4}$$

(4) $$S_{F}=\frac{S}{W}$$

The consistency of the semisolid formulations was evaluated using the pharmacopoeial penetrometry test [88]. The gel samples with a sufficient amount were prepared immediately after gelation and stored in the test container for 24 h at 25 ± 0.5 °C prior to testing. The gravity-driven penetrating object was released for 5 s, and the depth of penetration was measured in millimeters.

### 4.10. In Vitro Dissolution Test and Release Kinetics

The dissolution test was performed in a buffer medium with a pH = 5.5, simulating the physiological acidity of the skin. A sample (corresponding to 0.65 mg budesonide) was placed in a dialysis membrane (MW 10 000 Da) and introduced into a 50 mL acceptor phase tempered at 32 ± 0.5 °C at constant shaking. Aliquot samples were withdrawn at predetermined time intervals and replaced with fresh medium. The released drug amount was evaluated using the HPLC method. The chromatographic procedure was carried out with the HPLC system UltiMate Dionex 3000 SD, Chromeleon 7.2 SR3 Systems (Thermo Fisher Scientific Inc., Waltham, MA, USA). The separation was achieved with Column Luna (Phenomenex, Torrance, CA, USA) C18, 250 × 4.60 mm, particle size 5 μm, and a Diode Array Detector. The chromatographic conditions are as follows: mobile phase acetonitrile:methanol (70:30 *v*/*v*), flow rate 1.0 mL/min, and a wavelength of 254 nm. The amount was calculated based on a standard curve prepared in the concentration range of 3.5–10 µg/mL.

The drug release mechanism of the hydrogels was investigated by fitting the release profiles according to different release kinetic equations. Further, regression analysis was performed to evaluate the best fit.

## Figures and Tables

**Table 1:** Elastic (G′) and loss (G″) moduli and complex dynamic viscosity (η*) of plain methylcellulose (MC) and Pluronic F127 (F127) hydrogels and the corresponding hydrogels containing empty (NP-F127 and NP-MC) and drug-loaded nanoparticles (Bud-NP-F127 and Bud-NP-MC).

Sample	F127	NP-F127	Bud-NP-F127	MC	NP-MC	Bud-NP-MC
G′ (Pa)	22,960	26,900	27,570	69	240	191
G″ (Pa)	3037	1847	1757	108	231	185
η* (Pa.s)	3686	4292	4396	20	52	49

**Table 2:** Kinetic parameters of the in vitro drug release from the nanoparticles containing methylcellulose (Bud-NP-MC) and F127 (Bud-NP-F127) hydrogels.

Formulation	Zero Order	First Order	Higuchi	Korsmeyer-Peppas
	$Q_{t}\;=\;Q_{0}-k_{0}t$	$lnQ_{t}\;=\;lnQ_{0}-k_{1}t$	$Q_{t}\;=\;k_{H}t^{1/2}$	$\frac{M_{t}}{M_{\infty }}\;=\;k.t^{n}$
Bud-MC	R^2^ = 0.8870	R^2^ = 0.9875	R^2^ = 0.9874	R^2^ = 0.8909
	*k* = 13.895	*k* = −0.152	*k* = 37.678	*n* = 0.672
Bud-F127	R^2^ = 0.8598	R^2^ = 0.9848	R^2^ = 0.9850	R^2^ = 0.8344
	*k* = 14.567	*k* = −0.425	*k* = 40.073	*n* = 0.613
Bud-NP	R^2^ = 0.871	R^2^ = 0.9862	R^2^ = 0.9874	R^2^ = 0.8612
	*k* = 14.844	*k* = −0.2176	*k* = 40.619	*n* = 0.413
Bud-NP-MC	R^2^ = 0.9673	R^2^ = 0.9687	R^2^ = 0.9604	R^2^ = 0.9337
	*k* = 3.175	*k* = −0.021	*k* = 11.007	*n* = 0.268
Bud-NP-F127	R^2^ = 0.9599	R^2^ = 0.8673	R^2^ = 0.9089	R^2^ = 0.9125
	*k* = 8.309	*k* = −0.090	*k* = 28.133	*n* = 0.418

*Q*—amount of drug; *k*—rate constant; *t*—time; *n*—release exponent.

## Data Availability

The data are contained within the article.
